# An insulator defect detection method for transmission lines in complex weather conditions based on improved YOLOv8n

**DOI:** 10.1371/journal.pone.0356260

**Published:** 2026-08-26

**Authors:** Xiuyang Yuan, Zhiyu Shang, Jiaman Fang, Han Zhang, Yuhang Ou

**Affiliations:** 1 China Three Gorges University, Yichang, Hubei, China; 2 Guoke Nengchuang (Beijing) Technology Co., Ltd., Beijing, China; Swiss Federal Technology Institute of Lausanne, SWITZERLAND

## Abstract

Addressing challenges in UAV power line inspection—where insulator defect detection models are prone to environmental interference, insufficient feature representation, and difficulty balancing lightweight requirements—this study develops a lightweight image defect detection model that integrates high accuracy with strong robustness. An enhanced algorithm based on YOLOv8n is proposed. MobileNetV4 is adopted as the lightweight backbone, CBAM is introduced to enhance defect feature representation, ABIFPN is designed for multiscale bidirectional feature fusion, and SIoU is employed to improve localization accuracy. A multi-weather dataset containing 3,851 images of self-shattered and damaged insulators under rainy, snowy, foggy, overcast, and varying-exposure conditions was constructed using real and synthesized images. The dataset was divided into training, validation, and test sets at a ratio of 7:2:1. Across five independent experiments, the proposed model improved precision, recall, mAP@0.5, and mAP@0.5:0.95 by 2.43, 2.32, 2.43, and 5.47 percentage points, respectively, compared with the baseline. With a model size of only 7.01 MB, demonstrated better overall detection performance than YOLOv5n and YOLOv7-tiny. These results indicate its potential for UAV-mounted edge-based transmission-line inspection. However, some weather samples were synthetically generated, and more real-world data will be incorporated in future work.

## 1. Introduction

With the widespread application of Unmanned Aerial Vehicles (UAVs) in power line inspection, identifying line defects through image recognition has become a core issue in smart grid maintenance and management, with deep learning undoubtedly being the key to addressing this challenge [1–3]. Insulator defects are among the most common types of faults in transmission lines. However, constrained by the computational limitations of edge devices and the complex, variable conditions of field environments, the identifying features of defects often become less distinct, leading to missed detections or false positives [4–7]. Consequently, it is imperative to design a deep learning model suitable for UAV deployment that embodies both lightweight characteristics and strong robustness.

The YOLO series models [8], as representative single-stage detection methods in deep learning, are widely used for image defect detection of transmission line insulators. It should be noted that general insulator detection and insulator defect detection represent visual tasks at different levels of complexity. General insulator detection primarily aims to identify the locations and categories of complete insulators, whose object contours are relatively intact. In contrast, insulator defect detection requires the further identification of localized anomalies, such as self-shattering and physical damage. These defect regions usually occupy only a small proportion of the image and are often characterized by class imbalance, low contrast, partial occlusion, and visual similarities between defect textures and background elements such as towers, conductors, accumulated snow, and shadows. Consequently, insulator defect detection relies more heavily than general insulator detection on the preservation of fine-grained features, multiscale feature fusion, and the suppression of complex background interference. Reference [9] achieved lightweight modification of the YOLOv5 model by introducing GhostConv and C3Ghost modules into the backbone and neck networks, enhancing its capability for small object detection. However, in detection tasks involving complex backgrounds, model performance is determined not only by the network architecture but also by factors such as dataset distribution and training strategies. Compared with the C3 module used in YOLOv5, the C2f module employed in YOLOv8 offers architectural advantages in terms of feature reuse and gradient information propagation. Reference [10] modified the YOLOv8m model using CAM and CSO, improving its feature extraction capability for complex environments. Nevertheless, the resulting model has approximately 27.44M parameters, significantly larger than the 6.1M parameters of the YOLOv8n model, thus failing to meet the lightweight requirements for UAV deployment. Reference [11] improved the YOLOv8n model with a modified C2f module and an Adaptive Feature Pyramid Network (AFPN), balancing lightweight design and accuracy. However, when handling complex environments, its adaptive spatial feature fusion module may be contaminated by the complex background, leading to the propagation of noisy information to higher levels and reducing feature purity. Consequently, the robustness of this model is relatively weak. Although the aforementioned studies have made notable progress in image-based defect detection for transmission-line insulators, challenges remain under complex weather conditions. In conventional lightweight models, insulator defect features are easily obscured by background noise, resulting in insufficient feature extraction and multiscale feature fusion, and ultimately limiting detection accuracy.

Beyond insulator defect detection, related methods can be broadly categorized into three research directions: weather-robust detection, UAV-based edge inference, and small-object detection. Weather-robust detection methods typically employ learnable image enhancement or jointly optimize image enhancement and object detection to improve target visibility under degraded conditions such as fog and low illumination [12]. Research on UAV-based edge inference mainly adopts lightweight backbone networks, depthwise separable convolutions, and efficient feature fusion structures to balance detection accuracy, model complexity, and inference speed on embedded platforms [13]. Small-object detection methods enhance fine-detail representation by incorporating high-resolution detection heads, attention mechanisms, and multiscale feature fusion [14]. However, standalone image enhancement may introduce artifacts, excessive model compression may result in the loss of subtle defect features, and the addition of detection heads or complex feature fusion structures inevitably increases computational overhead. Therefore, for insulator defect inspection in complex environments, a unified lightweight solution capable of simultaneously achieving weather robustness, sensitivity to small defects, and efficient real-time onboard deployment remains lacking.

In practice, with the advancement of smart grids, deep learning techniques have been extensively applied in transmission line inspection processes, such as the detection of external intrusion threats [15] and the identification of multiple hardware components [16]. UAV-based inspection is an electromechanical system integrating perception, computation, communication, and actuation, and its operation is subject to multiple performance constraints. With the widespread adoption of UAVs and edge-computing devices, image-based defect detection models require further optimization, particularly to improve their adaptability to complex outdoor weather conditions while reducing the parameter count of deep learning models.

Therefore, addressing the challenges posed by complex weather conditions during UAV-based transmission line inspection, this paper proposes an image-based insulator defect detection method utilizing an improved YOLOv8n architecture. The main contributions of this work are as follows: (1) The strategic replacement of the original backbone network with MobileNetV4, which substantially reduces the computational burden and model size. (2) The integration of the Convolutional Block Attention Module (CBAM) to enhance the detection capability for small targets and mitigate interference from complex weather conditions. (3) The design of a novel ABiFPN neck structure by combining Selective Dimension Integration (SDI) with a Bidirectional Feature Pyramid Network (BiFPN). This addresses the limitations of traditional small object detection and endows the resulting feature pyramid with greater robustness. (4) The adoption of the SIoU loss function to optimize the YOLOv8n model. By incorporating considerations for the shape and orientation of insulators, it achieves higher accuracy in bounding box prediction. (5) By combining real-world images with image synthesis techniques, an insulator defect dataset covering complex weather conditions, including rain, snow, and fog, was constructed.(6) The NVIDIA Jetson Orin NX (16 GB) was selected as the target deployment platform. At an input resolution of 640 × 640 pixels, a model size of no more than 10 MB, an inference speed of at least 30 FPS, and a per-frame inference latency of no more than 33 ms were established as the engineering criteria for practical deployment validation.

## 2. Yolov8n algorithm: Principles and limitations

### 2.1. Principle of insulator image defect recognition using the YOLOv8n algorithm

The YOLOv8 algorithm, proposed by Ultralytics in 2023, is a real-time, single-stage object detection method. It is available in five versions with increasing parameter counts: YOLOv8n, YOLOv8s, YOLOv8m, YOLOv8l, and YOLOv8x. Among them, YOLOv8n is the lightweight version, offering the fastest speed and smallest size while maintaining satisfactory accuracy. The baseline model adopted in this study is the standard Ultralytics YOLOv8n detection architecture, with an input image size of 640 × 640 pixels. The model was configured to detect three categories: insulators, self-shattering defects, and damage defects. The official standard architecture contains approximately 3.16 million parameters and requires 8.9 GFLOPs, while the baseline weight file obtained through training in this study has a size of 6.04 MB. On the NVIDIA Jetson Orin NX (16 GB) platform, the baseline model achieved an inference speed of 185 FPS and a per-frame inference latency of 5.4 ms.

**Fig 1:**
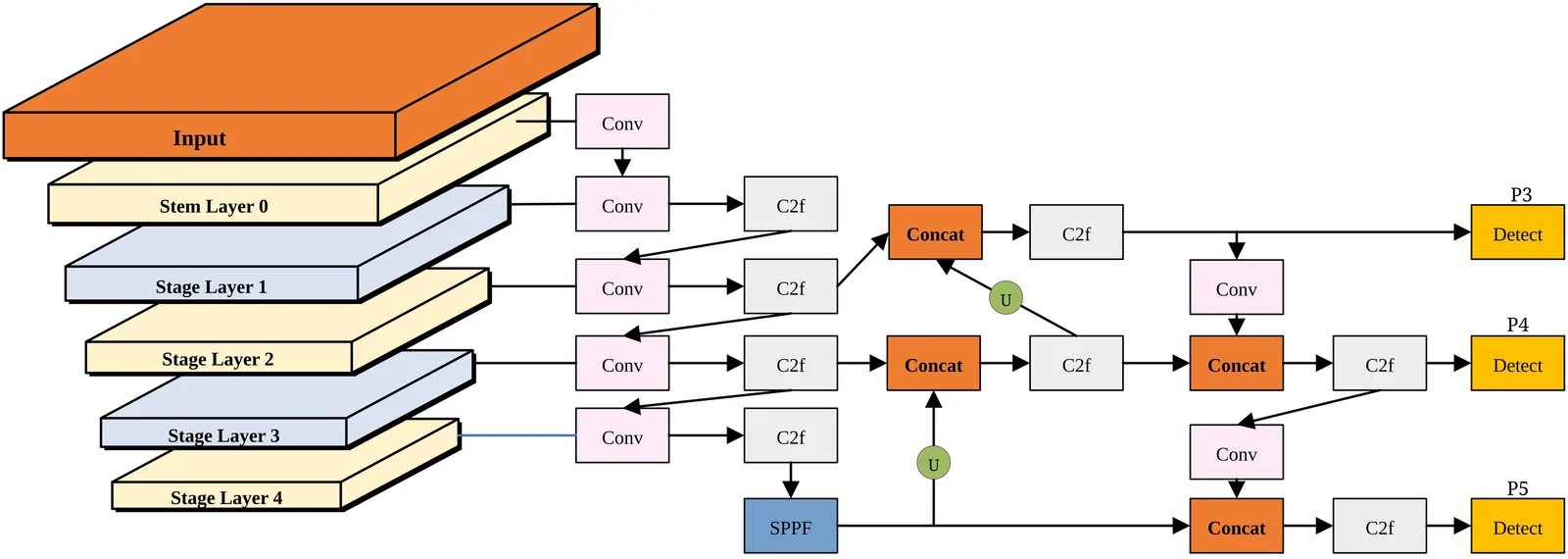
Schematic diagram of the YOLOv8n architecture.

The YOLOv8n architecture consists of three main components: the Backbone, the Neck, and the Head. The backbone network consists of Conv, C2f, and SPPF modules designed according to the cross-stage partial (CSP) principle. The Conv modules perform local edge and texture extraction as well as downsampling. The C2f modules improve gradient propagation through multibranch feature reuse, thereby facilitating the preservation of fine-grained characteristics such as fractured insulator sheds, notches, and self-shattered regions. The SPPF module enlarges the receptive field and incorporates contextual information from surrounding towers and conductors. However, successive downsampling may weaken the responses of low-contrast defects that occupy only a small proportion of the image. The neck network adopts a PAN-FPN-style architecture with top-down and bottom-up pathways. Upsampling and concatenation operations are used to integrate shallow localization details with deep semantic information, generating feature maps at three scales: P3/8, P4/16, and P5/32. Among them, the highest-resolution P3 feature map is the most critical for detecting small defects, whereas P4 and P5 provide complementary structural and contextual information. The detection head employs an anchor-free and decoupled prediction scheme. The classification branch outputs the class probabilities for insulators, self-shattering defects, and damage defects, while the regression branch predicts the locations and dimensions of the bounding boxes. During inference, predictions with low class-confidence scores are first removed according to a predefined confidence threshold. Non-maximum suppression (NMS) is then performed based on the intersection over union (IoU) between candidate boxes to eliminate duplicate detections of the same target while retaining high-confidence predictions. Therefore, the classification branch determines “what” the detected object is, the regression branch determines “where” it is located, the confidence threshold controls the reliability of candidate predictions, and NMS removes redundant predictions with substantial spatial overlap. A schematic diagram of the YOLOv8n architecture is shown in Fig 1 below.

As shown in Fig 1, Stem Layers 2–4 provide multilevel features from high-resolution details to high-level semantics. The top-down and bottom-up paths fuse these features, and the P3, P4, and P5 heads jointly produce the detection results. The P3 branch is particularly important for small defects. However, adverse weather and repeated downsampling may weaken or remove shallow defect features, which cannot be effectively recovered by simple concatenation and upsampling. This motivates the backbone replacement, attention mechanism, and improved multiscale feature fusion adopted in this study.

### 2.2. Limitations of the YOLOv8n algorithm for insulator defect detection in complex weather conditions and proposed improvements

**Fig 2:**
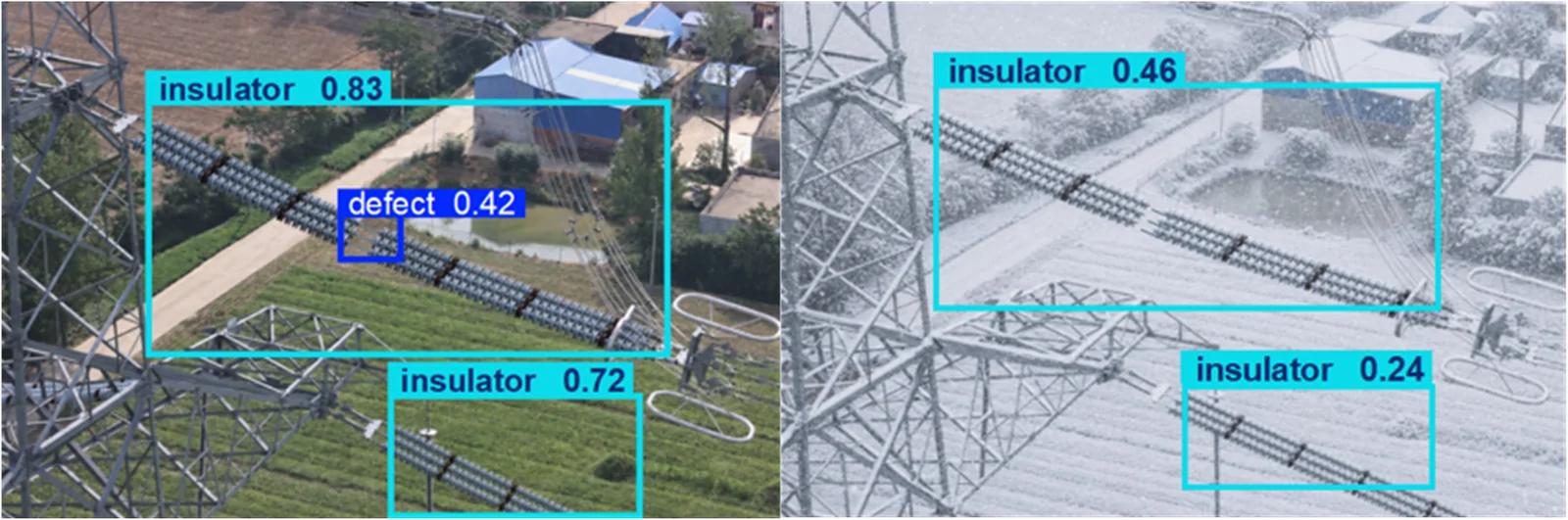
Comparative analysis of detection results by YOLOv8n under different weather conditions. The main limitation of YOLOv8n for insulator defect detection under complex weather conditions is its insufficient feature extraction capacity due to the restricted model size [9,18] Background noise, including rain streaks, snowflakes, and motion blur caused by UAV vibration, may resemble self-shattered or damaged regions in texture, edges, or grayscale intensity, thereby obscuring genuine defect features or generating false responses and ultimately leading to missed or false detections.

The YOLOv8n model deployed on UAV edge inspection devices, while lightweight, faces limitations due to its CSPDarkNet backbone network being constrained by computational capacity. This makes it susceptible to adverse weather conditions such as rain, snow, fog, strong illumination, and low light, which can cause insulator defect features to be obscured by background noise [17]. It will also be affected by non-weather-related degradation factors such as flight vibration, motion blur, focus deviation, lens dirt, scale changes, and complex background occlusion. Furthermore, the PANet in the neck network is prone to weather interference, resulting in low efficiency in feature pyramid fusion and weak correlations between features at different scales under complex weather conditions. When deep-level features are propagated to shallow layers via upsampling, the recovery of fine details becomes insufficient. The following Fig 2 shows a comparative analysis of the detection results obtained by the YOLOv8n model for insulator defects on transmission lines under sunny and snowy conditions.

In addition to the model architecture, annotation quality may also limit detection performance. Under rain or snow occlusion, fog, overexposure, and long-distance small-object conditions, defect boundaries are often unclear, leading to inter-annotator inconsistency. Loose or tight bounding boxes, missing or incorrect labels, and class confusion can introduce label noise and impair classification and localization learning. Future dataset construction should therefore adopt standardized annotation guidelines, independent cross-checking by two annotators, arbitration of disputed samples, and quality grading for low-visibility images to reduce annotation bias in training and evaluation.

To address these issues, the following im-provements to the YOLOv8n model are proposed: (1) The backbone network should be modified to enhance its robustness against weather variations by incorporating depthwise separable convolutions, while preserving its lightweight design; (2) The neck network requires a stronger resistance to background noise to improve feature extraction; (3) The model’s multi-scale fusion capability must be enhanced to reduce the attenuation of feature information during propagation, thereby increasing overall detection accuracy.

## 3. An improved Yolov8n-based algorithm for insulator defect identification

### 3.1. MobileNetV4 backbone network

**Fig 3:**
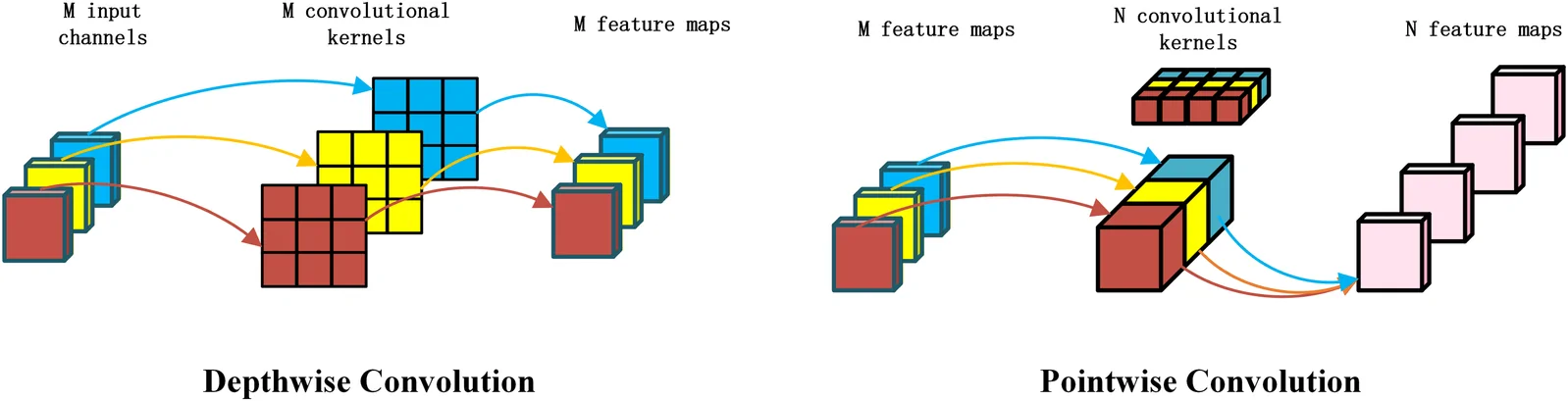
Schematic diagram of the convolutional principles in the MobileNetV4 backbone network. Compared to the computationally expensive CSPDarknet backbone network, adopting Mobile-NetV4 as the backbone enables a more lightweight model with faster inference speed. The reduction in parameter count also reserves computational budget for integrating additional modules, preventing the overall improved model from becoming redundant.

MobileNet is a lightweight Convolutional Neural Network (CNN) [19] specifically designed for mobile and edge devices. MobileNetV4, as the latest iteration of this architecture, employs a combination of depthwise and pointwise convolutions, reducing computational cost to approximately one-ninth of that required by standard convolution operations [20]. Fig 3 illustrates the con-volutional scheme of the MobileNetV4 model.

It should be noted that replacing the backbone involves a trade-off between model lightweighting and feature representation capability. MobileNetV4 uses depthwise separable convolutions to reduce parameters and computation, but its cross-channel interaction and high-level semantic representation may be weaker than those of CSPDarkNet. Excessive lightweighting may therefore result in the loss of subtle defect features under low-contrast, long-distance, heavily occluded, or extreme-weather conditions. Accordingly, this study does not simply pursue a smaller model. Instead, CBAM and ABIFPN are introduced to compensate for the representation of critical regions and multiscale detail fusion, while the benefits of backbone replacement are comprehensively evaluated in terms of detection accuracy, model size, and inference speed.

On the other hand, although the MobileNetV4 backbone features fewer parameters and lower computational costs, it incorporates an increased number of channels and employs the superior Hardswish activation function [21]. These enhancements enable the network to better address the challenges posed by complex weather conditions overall. The Depthwise Convolution structure [22] adopted by this network offers unique anti-interference advantages. By decoupling feature extraction into spatial feature acquisition and channel feature fusion, the convolution process acts analogously to a low-pass filter in the spatial frequency domain when dealing with complex weather environments, effectively filtering out high-frequency noise induced by such conditions. Furthermore, while the network introduces an attention mechanism absent in CSPDarknet, its inherent lightweight Squeeze-and-Excitation (SE) attention mechanism [23] can only suppress noisy channels to a limited extent. In real-world scenarios, background environments caused by complex weather are often non-uniform. Under such circumstances, the SE attention mechanism assigns uniform weights to different image regions, failing to achieve targeted attention enhancement for specific areas. Consequently, a more sophisticated attention mechanism is required to compensate for these limitations.

### 3.2. CBAM attention mechanism

The Convolutional Block Attention Module (CBAM) is a simple yet effective attention mechanism widely adopted in various convolutional neural networks (CNNs). Traditional research on enhancing CNN performance has primarily focused on increasing network depth, width, or cardinality. In contrast, the advent of attention mechanisms enables models to learn to focus on salient regions while suppressing non-essential features.

**Fig 4:**
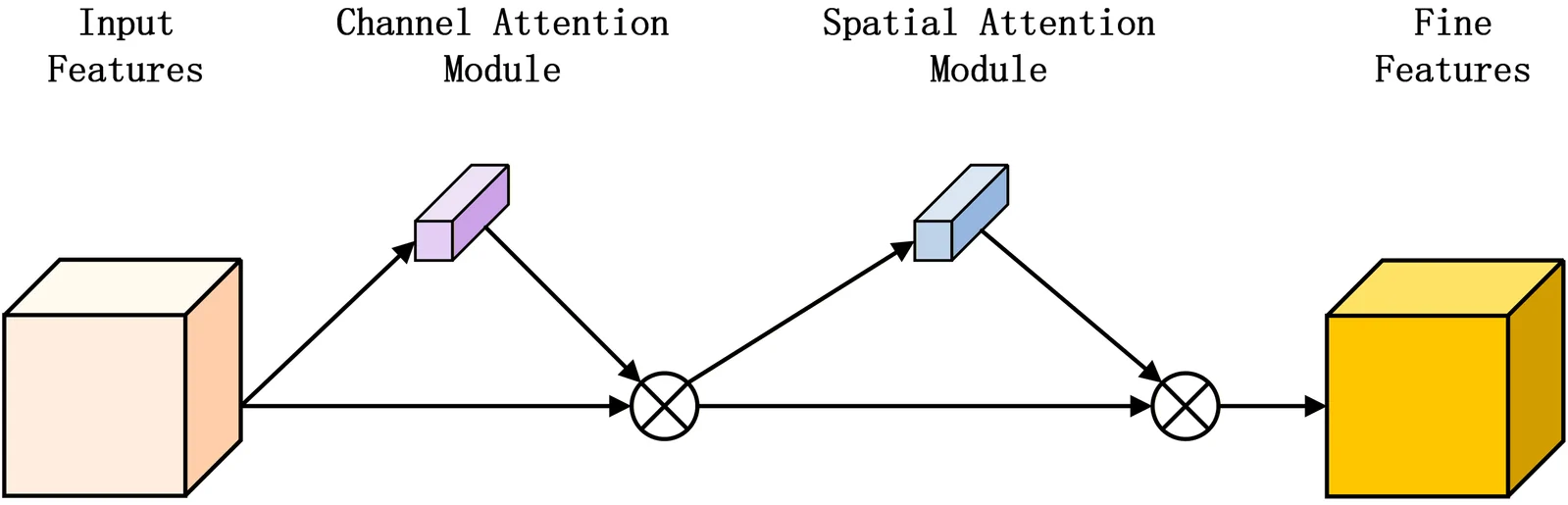
Schematic diagram of the CBAM attention mechanism. When addressing background interference caused by complex weather conditions, the CBAM attention mechanism computes relationships across spatial channels in insulator images to generate corresponding spatial weight maps. These maps effectively highlight potential defect locations for subsequent detection heads, enabling more precise defect boundary determination in these regions and enhancing the model’s overall sensitivity to minute defects.

During image feature extraction, convolutional operations primarily extract informative features by blending cross-channel and spatial information. The CBAM mechanism consists of two sequential sub-modules: a channel attention module and a spatial attention module. The channel attention module guides the model to emphasize critical features (e.g., the insulator’s structure, defect characteristics), whereas the spatial attention module directs the model’s focus to significant regions within the image (e.g., potential insulator locations, areas with distinct features). Schematic diagram of the CBAM attention mechanism is shown in Fig 4 below.

The CBAM module is computationally efficient by design. Its use of global average pooling and max pooling operations introduces no additional parameters or significant computational overhead. Furthermore, its high compatibility enables seamless integration into various convolutional neural network architectures.

The CBAM module complements the previously mentioned SE attention mechanism, addressing the “one-size-fits-all” limitation in MobileNetV4’s feature weighting approach. Simultaneously, it provides more refined and accurate insulator feature information for the subsequent feature pyramid network.

### 3.3. ABIFPN neck structure

The Selective Dimensional Integration (SDI) multi-level feature fusion module serves as an adaptive feature selection and enhancement mechanism. When UAVs conduct power inspections under complex weather conditions (e.g., rain, snow, fog), the image quality of insulators degrades, introducing substantial irrelevant information and interference into the convolutional neural network. SDI not only filters out this extraneous information but also effectively integrates high-level semantics (indicating the presence of a defective insulator) with low-level details (such as fracture locations), thereby enhancing the model’s capability to detect both subtle and structural defects.

**Fig 5:**
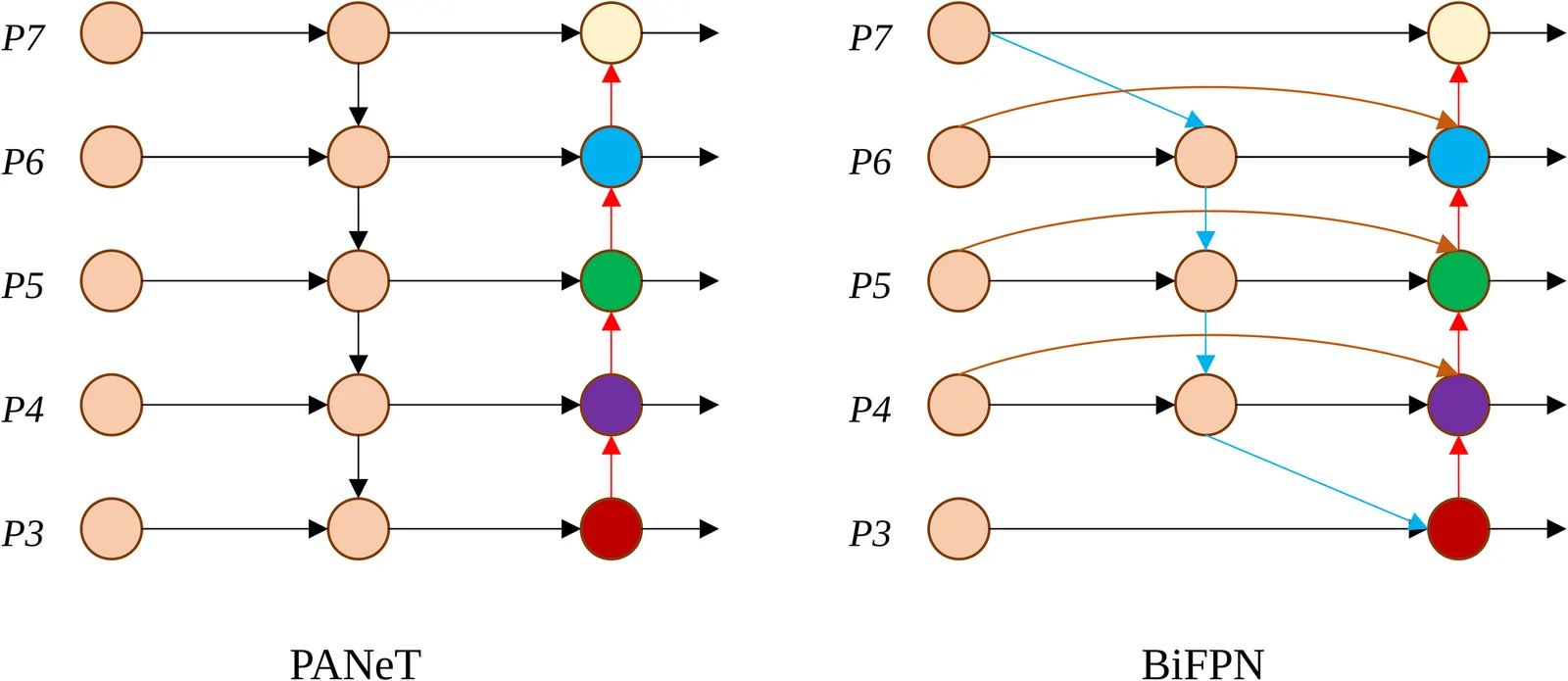
Comparison of feature network fusion between PANet and BiFPN.

The Bidirectional Feature Pyramid Network (BiFPN [24]) represents an optimization over the original PANet neck structure. Leveraging its characteristic weighted feature fusion capability, BiFPN performs weighted integration of multi-scale insulator features based on their relative importance and contribution, thereby ensuring more effective robustness in the fused feature representations. Comparison of feature network fusion between PANet and BiFPN is shown in Fig 5 below.

**Fig 6:**
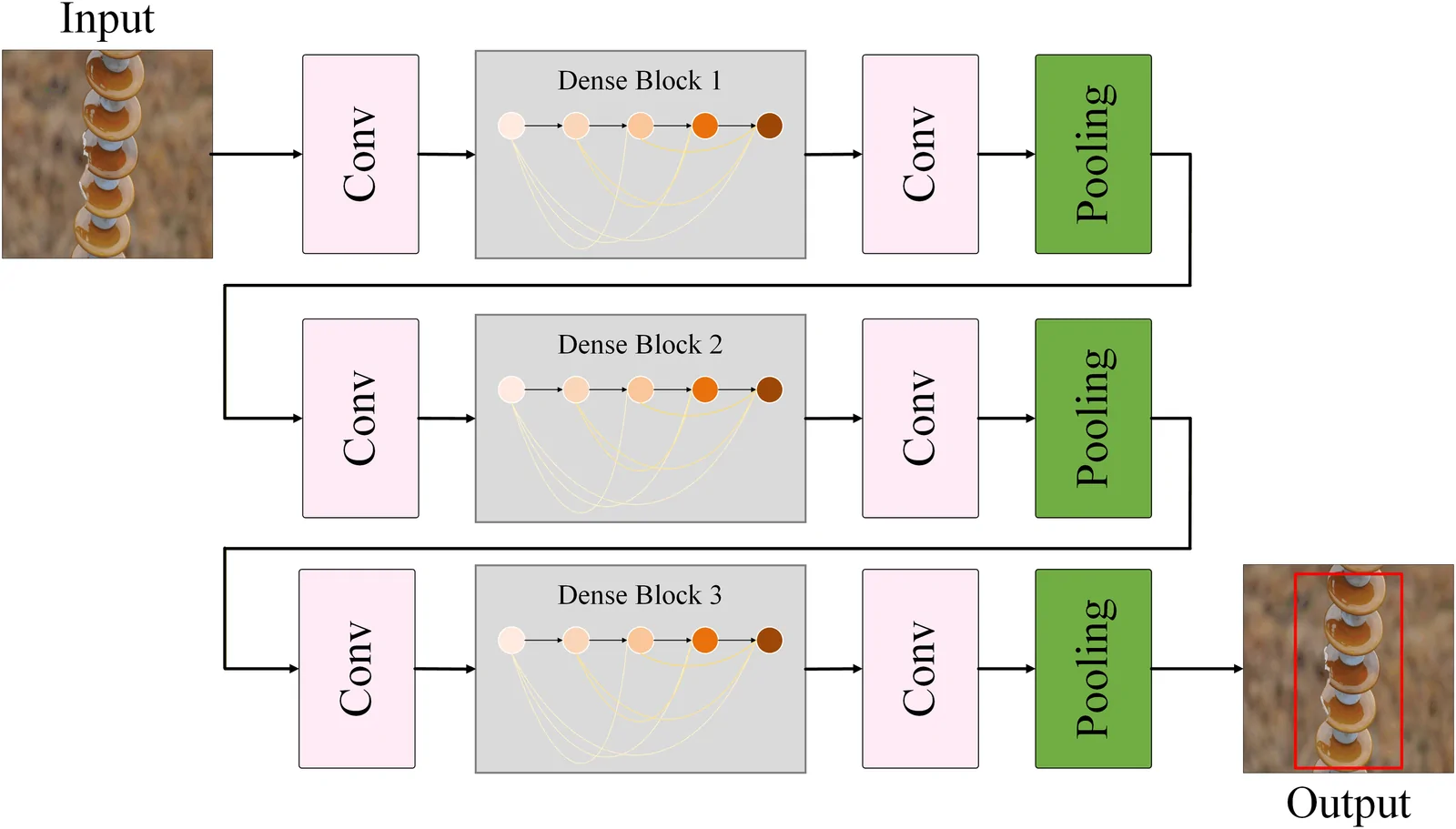
Architecture of the ABIFPN structure.

The ABIFPN neck structure represents a deep integration of SDI and BiFPN at the mechanistic level. Following BiFPN’s bidirectional feature fusion, the embedded SDI module guides the network to prioritize deep-level semantics while enhancing shallow-level features. This integration introduces greater selectivity and purposefulness into the bidirectional fusion process, yielding higher-quality fused features for insulators and consequently improving the model’s precision in defect identification. Architecture of the ABIFPN structure is shown in Fig 6 below.

### 3.4. SIoU loss function

SIoU (Scylla-IoU) is a loss function used in object detection to improve bounding box regression. Compared to traditional loss functions (e.g., IoU, GIoU, CIoU), it incorporates the concept of directionality to achieve more stable convergence and higher accuracy. The regression process of the SIoU loss function consists of four components:

(1) Angle Cost Λ

Loss Function


$$\begin{array}{c} \Lambda =1-2*{sin}^{\text{2}}(arcsin(x)-\frac{\pi }{\text{4}}) \end{array}$$ (1)

where


$$\begin{array}{c} x=arcsin\left(\frac{{\text{c}}_{\text{h}}}{\sigma }\right)=\alpha \end{array}$$ (2)

$$\begin{array}{c} \sigma =\sqrt{{\left(b_{c_{x}}^{gt}-b_{c_{x}}\right)}^{\text{2}}+{\left(b_{c_{y}}^{gt}-b_{c_{y}}\right)}^{\text{2}}} \end{array}$$ (3)

$$\begin{array}{c} c_{h}=max\left(b_{c_{y}}^{gt},b_{c_{y}}\right)-min\left(b_{c_{y}}^{gt},b_{c_{y}}\right) \end{array}$$ (4)

**Fig 7:**
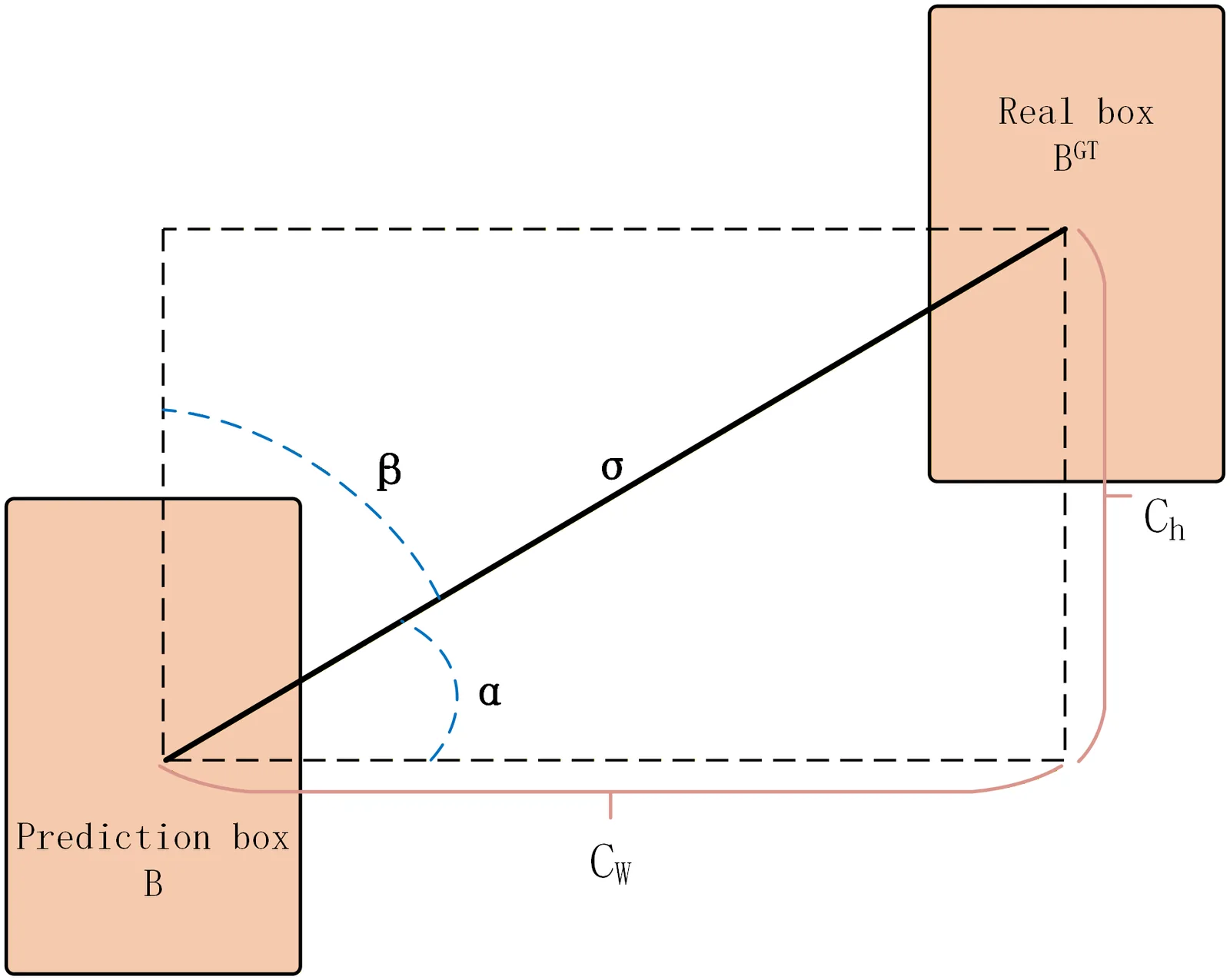
Schematic diagram of the angular cost in SIoU.

In the formula, σ represents the distance between the centers of the predicted and ground truth bounding boxes; α represents the angle between the centers of the predicted and ground truth bounding boxes; $c_{h}$ represents the height difference between the centers of the predicted and ground truth bounding boxes; $\left(b_{c_{x}}^{gt},b_{c_{y}}^{gt}\right)$ denotes the ground truth bounding box center coordinates; $\left(b_{c_{x}},b_{c_{y}}\right)$ denotes the predicted bounding box center coordinates. The Schematic Diagram of the Angular Cost in SIoU is shown in Fig 7.

(2) distance cost Δ

$$\begin{array}{c} \Delta =\sum_{t=x,y}\left(1-e^{-\gamma p_{t}}\right)=2-e^{-\gamma p_{x}}-e^{-\gamma p_{y}} \end{array}$$ (5)

where


$$\begin{array}{c} \rho_{x}=\text{(}\frac{b_{c_{x}}^{gt}-b_{c_{x}}}{c_{w}}{\text{)}}^{\text{2}} \end{array}$$ (6)

$$\begin{array}{c} \rho_{y}=\text{(}\frac{b_{c_{y}}^{gt}-b_{c_{y}}}{c_{h}}{\text{)}}^{\text{2}} \end{array}$$ (7)

$$\begin{array}{c} \gamma =2-\Lambda \end{array}$$ (8)

**Fig 8:**
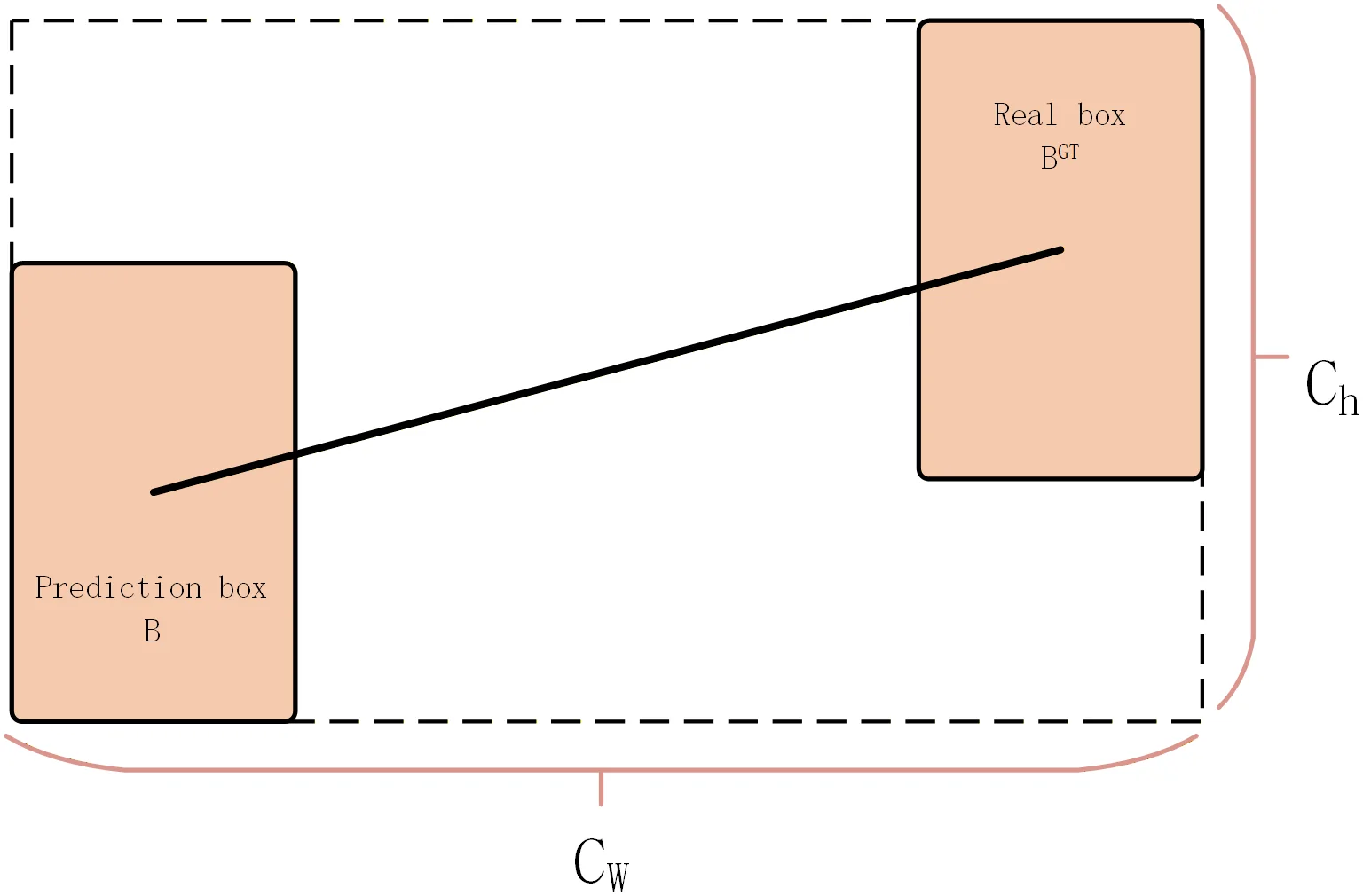
Schematic diagram of the distance cost in SIoU loss function.

In the formula, $c_{h}$ and $c_{w}$ represent the height and width of the minimum bounding rectangle of the ground truth box and the predicted box, respectively. The schematic diagram of the distance cost in the SIoU loss function is shown in Fig 8.

(3) Shape Cost Ω

$$\begin{array}{c} \Omega =\sum_{t=w,h}{\left(1-e^{-\omega_{t}}\right)}^{\theta } \end{array}$$ (9)

where


$$\begin{array}{c} \omega_{w}=\frac{\left|w-w^{gt}\right|}{max\left(w,w^{gt}\right)} \end{array}$$ (10)

$$\begin{array}{c} \omega_{h}=\frac{\left|h-h^{gt}\right|}{max\left(h,h^{gt}\right)} \end{array}$$ (11)

In the formula, (w,h) represent the width and height of the predicted bounding box; $\left(w^{gt},h^{gt}\right)$ represent the width and height of the ground truth bounding box; θ denotes the weight assigned to the shape loss component.

(4) IoU Cost

$$\begin{array}{c} I\text{o}U=\frac{Intersection\text{\textbackslash{}hspace\{1em}}{A}\}Union\text{\textbackslash{}hspace\{1em}B\} \end{array}$$ (12)

The final SIoU loss function is formulated as:


$$\begin{array}{c} L{\text{oss}}_{SIoU}=1-IoU+\frac{\Delta +\Omega }{\text{2}} \end{array}$$ (13)

**Fig 9:**
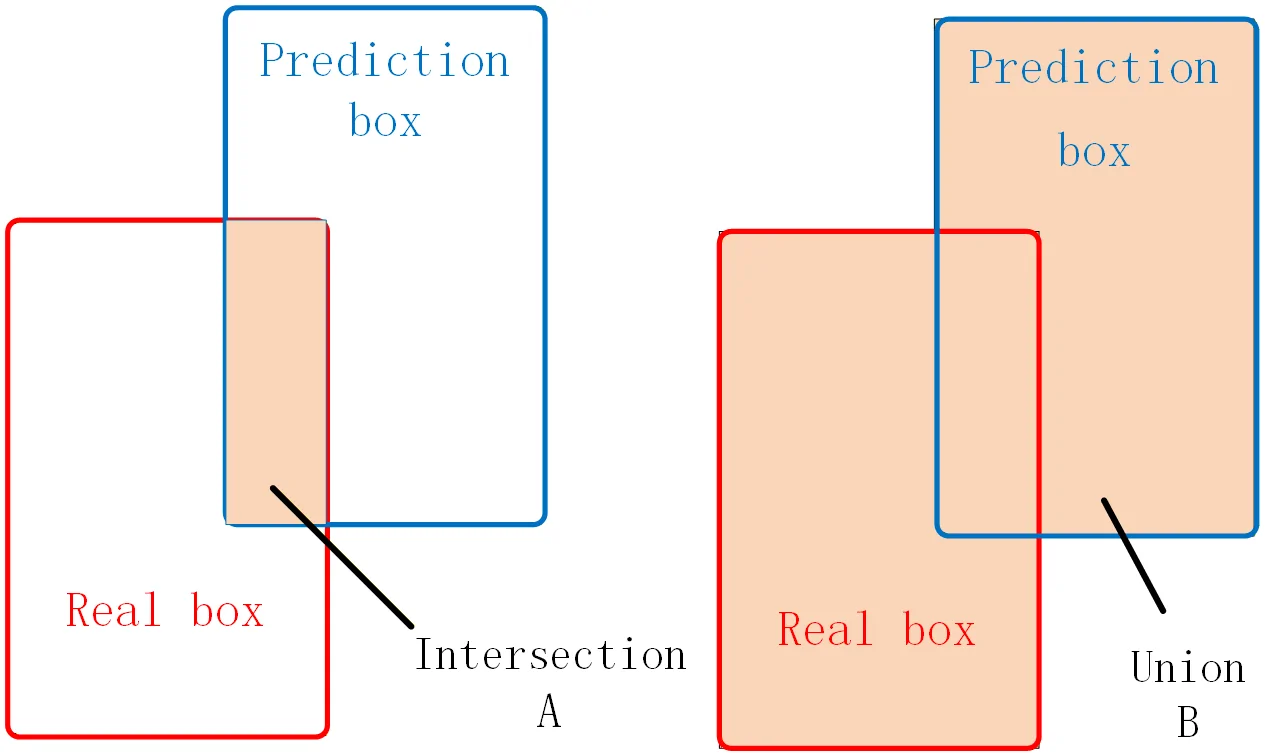
Schematic diagram of IoU loss.

The SIoU loss function facilitates superior bounding box regression through its directional awareness and shape constraints, leading to more precise insulator localization. When addressing complex weather conditions, its directional perception capability enables the model to maintain valid geometric reasoning even with obscured insulator features. The Schematic Diagram of IoU Loss is shown in Fig 9.

### 3.5. An improved YOLOv8n-based algorithm for insulator defect identification

**Fig 10:**
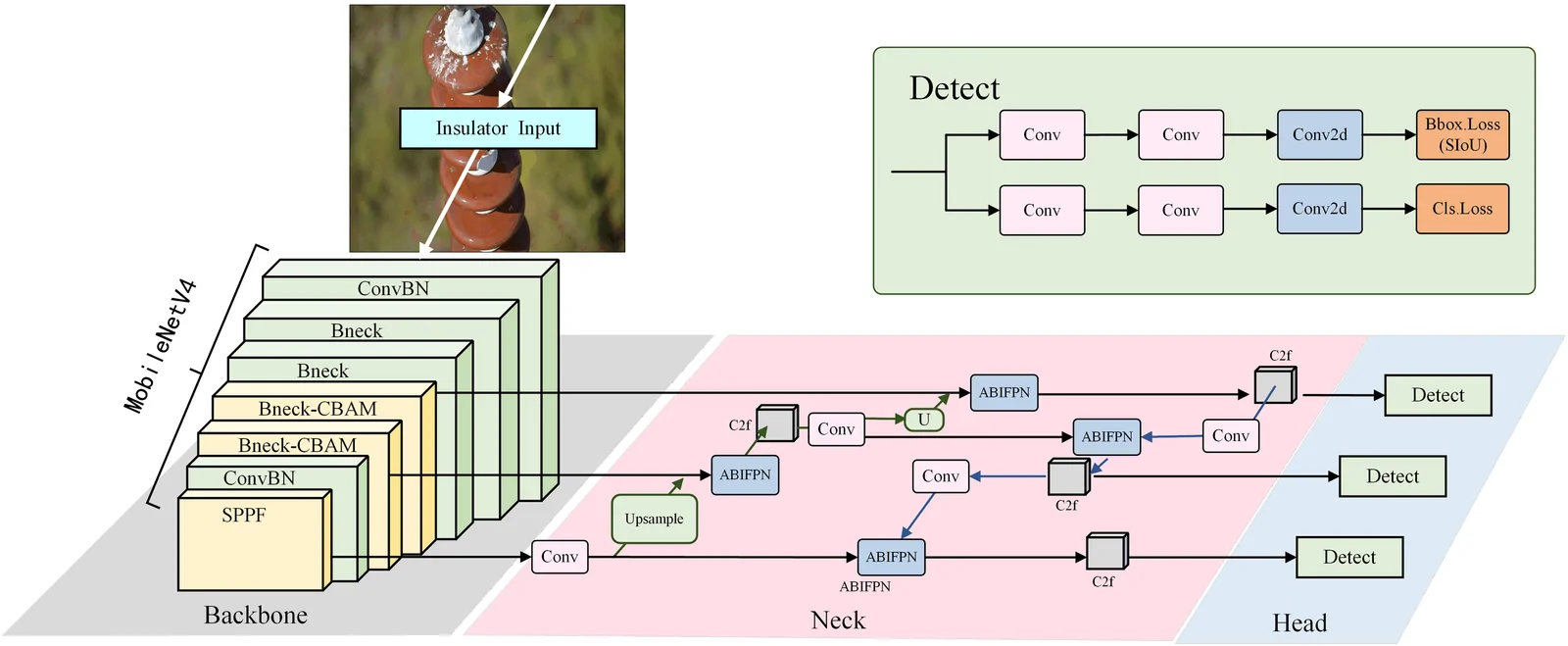
Schematic diagram of the improved YOLOv8n architecture.

The architecture of the improved YOLOv8n algorithm is illustrated in Fig 10. It employs MobileNetV4 as the backbone network with an integrated CBAM attention module, adopts the ABIFPN multi-scale feature fusion mechanism for the neck structure, and utilizes the SIoU loss function during training to obtain detection results.

The Improve the overall architecture and feature scale of the model have been presented in Table 1. The training and inference processes are summarized as follows. During training, augmented and normalized images are sequentially processed by the MobileNetV4-CBAM backbone, ABIFPN neck, and P3/P4/P5 detection heads. Classification loss, distribution focal loss, and SIoU loss are then calculated for backpropagation, and the best validation weights are saved. During inference, the same preprocessing and a single forward pass are performed, followed by confidence filtering and NMS to obtain the final classes, locations, and confidence scores.

**Table 1:** Improve the overall architecture and feature scale of the model.

Phase	Output Scale	Core Module	Main Function
Input	640 × 640 × 3	Resize, normalization	Uniformly input the size and pixel distribution
Stem	320 × 320(/2)	Conv-BN-HardSwish	Extract edges and basic textures
Stage 2	160 × 160(/4)	MobileNetV4 Block	Maintain local information with high resolution
C3/ P3	80 × 80(/8)	MobileNetV4 + CBAM	Assume the detection of minor defect details
C4/ P4	40 × 40(/16)	MobileNetV4 + CBAM	Integrate local structure with mid-level semantics
C5/ P5	20 × 20(/32)	MobileNetV4 + SPPF	Provide high-level semantics and context
Neck	P3, P4, P5	ABIFPN(BiFPN + SDI)	Bidirectional weighted fusion and noise suppression
Head	80 × 80/ 40 × 40/ 20 × 20	Anchor-free decoupled detection head	Output category probabilities and bounding boxes
Training loss	—	SIoU bounding box loss	Improve direction perception and positioning accuracy

Because MobileNetV4 and ABIFPN replace the original YOLOv8n backbone and neck, the standard YOLOv8n weights cannot be strictly loaded into the entire network. Only parameters with matching names and tensor dimensions are transferred. MobileNetV4 may use ImageNet-pretrained weights of the same architecture, whereas CBAM and ABIFPN are randomly initialized. SIoU only modifies the training loss and does not affect the inference weight format.

For a 640 × 640 input, the P3, P4, and P5 feature maps are 80 × 80, 40 × 40, and 20 × 20, respectively, with P3 primarily responsible for small-defect detection. Although adding a P2/4 head could preserve finer details, it would increase computation, memory consumption, and sensitivity to weather-related noise. Since the current P3/P4/P5 heads satisfy the accuracy and real-time requirements on the Jetson Orin NX, P2 is reserved for future ablation studies on smaller defects.

MobileNetV4 was selected over GhostNet and ShuffleNetV2 for its balance between lightweight design and feature representation. CBAM introduces spatial attention beyond the channel-only modeling of SE and ECA, ABIFPN combines bidirectional fusion with SDI-based feature selection, and SIoU further considers the direction between box centers compared with CIoU. As not all alternatives were repeatedly trained under identical settings, inconsistent quantitative comparisons were omitted to ensure fairness.

## 4. Experimental setup

### 4.1. Dataset construction

This study combines the CPLID dataset [25] with the IDID dataset [26]. After removing duplicate or near-duplicate images and severely defocused or motion-blurred images in which the insulators or defects could not be reliably identified, 987 normal-insulator images were retained, 362 self-shattered insulator images, and 625 damaged insulator images were obtained. To enhance data diversity, various augmentation techniques were applied to insulator images of different scales, including brightness adjustment, rotation, and translation. Image augmentation was performed for different weather and imaging conditions, including rain, snow, fog, cloudy weather, and exposure variations. The seven augmentation types generated 409, 409, 409, 409, 409, 409, and 410 images, respectively. Combined with the 987 original images, the expanded dataset contained 3,851 images and was divided into training, validation, and test sets at a ratio of 7:2:1. Although this approach expands complex-environment samples from limited real data, synthetic images may differ from the actual imaging distribution under extreme weather conditions. The data augmentation and weather simulation settings is shown in Table 2.

**Table 2:** Data augmentation and weather simulation settings.

Type	Implementation and Parameters
Rotation	Angle range: −10° to 10°; bounding boxes transformed accordingly
Translation/Scaling	Translation factor: 0.1; scale factor: 0.2; bounding boxes transformed accordingly
Rain	Rain-streak density, length, angle, blur kernel, and opacity
Snow	Particle size, density, number of depth layers, blur kernel, and opacity
Fog	Atmospheric light A, scattering coefficient β, and transmittance
Cloudy	Gamma, brightness, contrast, and saturation
Exposure	Exposure gain, gamma, and local-mask ratio

Object scales were calculated for 640 × 640 inputs, with small, medium, and large objects accounting for approximately 60%, 30%, and 10%, respectively. The median defect-box area represented 0.21% of the full image and 6.5% of the corresponding insulator box. At the image level, the dataset contained 362 self-shattering samples and 625 damage samples; thus, the former accounted for approximately 57.9% of the latter, indicating moderate class imbalance. Minority-class compensation was applied only to the training set through targeted augmentation or weighted sampling to increase the occurrence of self-shattering defects. The validation and test sets retained their natural distributions without resampling.

### 4.2. Experimental configuration

The experiments were conducted on a Windows 11 64-bit operating system with the following hardware configuration: an Intel(R) Core(TM) Ultra 9 275HX CPU (2.70 GHz) and an NVIDIA GeForce RTX 5070 Ti GPU. The deep learning framework used was PyTorch 2.4.1, with Python 3.10 and CUDA 13.0 as the software environment.

The training parameters for the model were set as follows: 200 epochs, an image size (imgsz) of 640 × 640 pixels, SGD optimizer, batch size of 32, and a learning rate (lrf) of 0.01, momentum = 0.937, weight decay = 0.0005, and warm-up epochs = 3. Color augmentation parameters were set to *hsv*_*h*_ = 0.015, *hsv*_*s*_ = 0.7, and *hsv*_*v*_ = 0.4, while geometric augmentation parameters were set to degrees = 10, translate = 0.1, and scale = 0.2.

### 4.3. Evaluation metrics

This paper employs Precision, Recall, F1-score, Average Precision (AP), and mean Average Precision (mAP) to evaluate model accuracy. Specifically, mAP@0.5 denotes the mAP calculated at an IoU threshold of 0.5, representing a relatively lenient criterion. Meanwhile, mAP@0.5-0.95 refers to the average mAP over IoU thresholds from 0.5 to 0.95 with a step size of 0.05, serving as a more stringent metric that better reflects the model’s precise detection capability. The calculation formulas are as follows:


$$\begin{array}{c} AP=\int_{\text{0}}^{\text{1}}P\left(r\right)dr \end{array}$$ (14)

$$\begin{array}{c} mAP=\frac{\text{1}}{n}\sum_{i=0}^{n}AP_{\left(i\right)} \end{array}$$ (15)

$$\begin{array}{c} Precision=\frac{TP}{TP+FP} \end{array}$$ (16)

$$\begin{array}{c} Recall=\frac{TP}{TP+FN} \end{array}$$ (17)

$$\begin{array}{c} F_{\text{1}}=\frac{2\times Precision\times Recall}{Precision+Recall} \end{array}$$ (18)

In the formula, *P(r)* represents the smoothed Precision-Recall (PR) curve; *n* denotes the number of classes in the dataset; *TP* indicates the number of correctly identified insulators; *FN* refers to the number of undetected insulators, *FP* refers to the number of falsely detected insulators. *All experimental results were obtained from five independent runs, with confidence intervals below 2% and standard deviations below 1%.*

## 5. Experiments and results

### 5.1. Ablation study

To validate the effectiveness of each improvement module in our modified YOLOv8n model, under the above experimental conditions, Experiments 1–3 were conducted as independent ablations, whereas Experiments 4–7 employed a progressive combination strategy. The Table 3 below presents the results of the ablation experiments.

**Table 3:** Ablation study results.

Group	MobileNetV4	CBAM	ABIFPN	SIOU	Precision	Recall	mAP@0.5	mAP@0.5-0.95	Weight	F1
Baseline					94.90%	94.69%	94.95%	73.92%	6.04 M	94.79%
1		√			96.59%	96.44%	96.52%	75.02%	6.13M	96.52%
2			√		97.58%	95.88%	96.61%	75.59%	6.41M	96.72%
3				√	96.46%	95.69%	95.99%	75.83%	5.96M	96.07%
4	√				96.67%	94.98%	97.16%	75.84%	4.03M	95.81%
5	√	√			96.94%	96.26%	96.40%	75.35%	4.33M	96.60%
6	√	√	√		97.32%	96.26%	96.98%	78.73%	7.01M	96.79%
7	√	√	√	√	97.33%	97.01%	97.38%	79.39%	7.01M	97.17%

As shown in the ablation experiment results, the four improvement measures in this paper, whether applied individually or in combination, bring significant performance enhancements to the original YOLOv8n model, though each focuses on different aspects: Using the MobileNetV4 backbone network alone improves both the accuracy and average precision of the model by nearly 2% each, while reducing the model size by nearly 2MB; the CBAM attention mechanism improves the model’s recall by nearly 2%; the ABIFPN structure achieves the highest precision when used individually, with an improvement of 2.68%; The use of SIoU loss yielded slight improvements across all performance metrics, with an increase of nearly 2 percentage points in mAP@0.5–0.95, indicating that SIoU provides a modest but consistent gain.

For the combined improvements, the effects of progressively integrating the four modules are as follows: Configuration 5 augments the MobileNetV4 backbone with the CBAM attention mechanism, addressing the significant Recall deficiency observed when using MobileNetV4 alone. However, this combination leads to a decrease in average precision, falling short of expectations. Configuration 6 incorporates the ABIFPN module into the neck structure, increasing both Precision and mAP@0.5 by 0.5%, while mAP@0.5-0.95 rises from 75.35% to 78.73%, a 3.38% improvement. Configuration 7 employs SIoU as the loss function, resulting in a nearly 1% increase in Recall and a 0.5% gain in mAP@0.5-0.95 for the final optimized model.

Compared to the original model, the improved YOLOv8n model achieves a 2.43% increase in Precision, a 2.32% improvement in Recall, a 2.43% gain in mAP@0.5, a 5.47% enhancement in mAP@0.5-0.95, and a 2.38% boost in F1-score, representing an overall performance improvement of approximately 3%.

**Fig 11:**
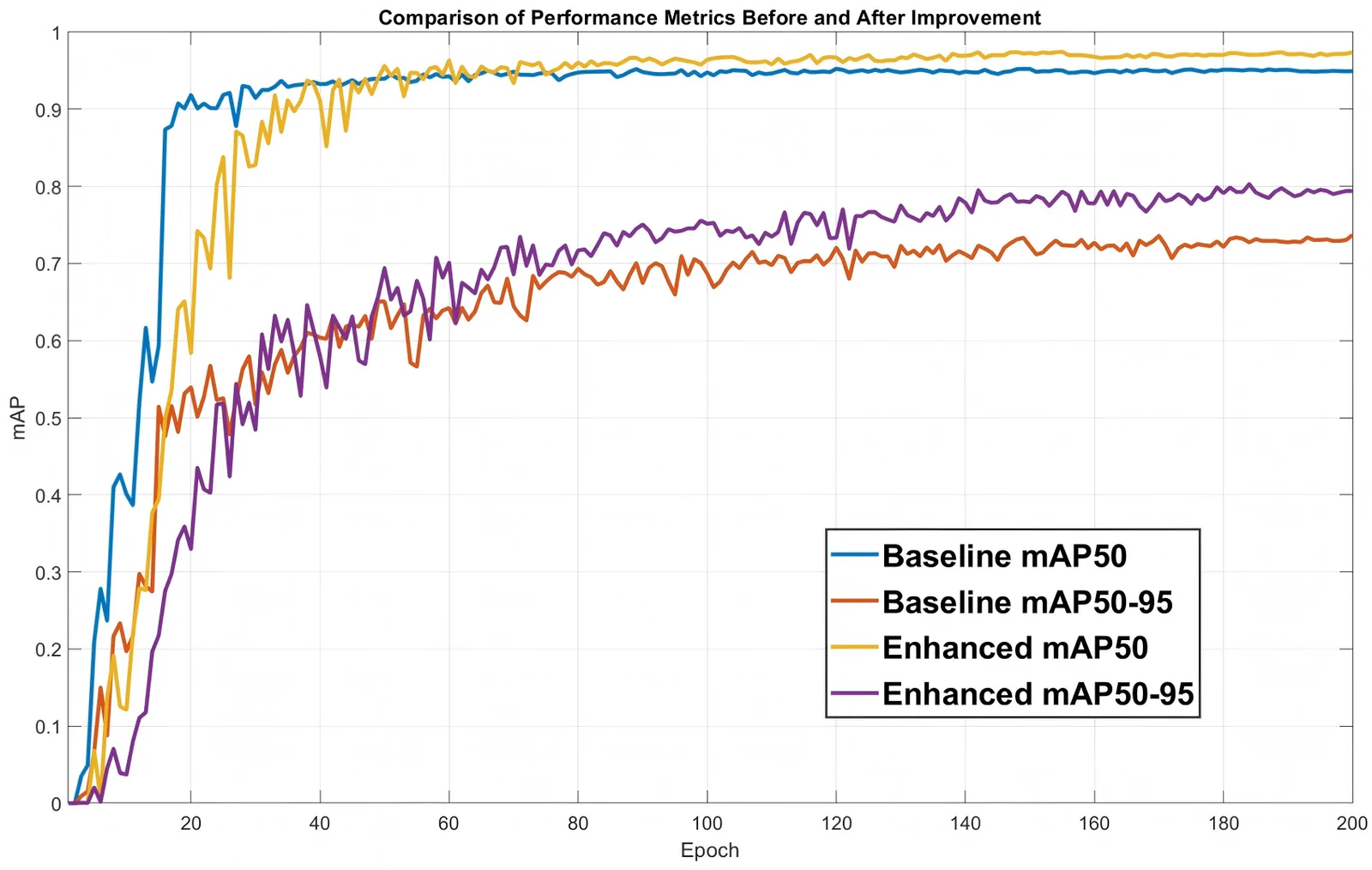
Comparison of mAP values before and after improvement. In Fig 11, the x-axis represents the epoch, and the y-axis represents mAP on a scale of 0–1. The blue and orange curves denote the baseline model’s mAP@0.5 and mAP@0.5–0.95, respectively, while the yellow and purple curves represent those of the improved model. Only validation mAP curves are presented; training and validation losses, precision, and recall are not included. Therefore, overfitting cannot be assessed from this figure alone.

The baseline YOLOv8n converges faster, becoming stable after approximately 20 epochs. After 50 epochs, however, the improved model outperforms the baseline in both mAP@0.5 and mAP@0.5–0.95. This indicates that the improved model requires approximately 30 additional epochs to reach stable performance. Nevertheless, both models were trained for the same 200 epochs, so no increase in the total training duration was required, while the improved model achieved higher final mAP values (Fig. 11).

### 5.2. Comparative experiments

To validate the effectiveness of our improved YOLOv8 algorithm, comparative experiments were conducted using other mainstream deep learning models (including YOLOv5n, YOLOv8n, Faster-RCNN, etc.) on our constructed insulator defect dataset. The comparative results of different algorithms are presented in Table 4 below:

**Table 4:** Comparative experimental results.

Model	Precision	Recall	map@0.5	map@0.5-0.95	F1	Weight
YOLOv8n	94.90%	94.69%	94.95%	73.92%	94.79%	6.04 M
YOLOv5n	94.50%	95.47%	95.26%	72.66%	94.98%	**5.03 M**
YOLOv12n [27]	93.61%	94.44%	95.01%	73.43%	94.02%	5.27 M
YOLOv6n	94.54%	94.87%	95.43%	74.79%	94.71%	8.29 M
YOLOv10n [28]	90.23%	94.26%	94.20%	74.02%	92.20%	5.49 M
YOLOv11n [12]	94.48%	95.49%	94.98%	73.60%	94.98%	5.22M
YOLOv7-tiny	94.40%	94.82%	95.91%	67.35%	94.61%	12.04 M
YOLOr-csp	92.95%	94.79%	95.76%	68.79%	93.86%	18.26 M
YOLOv4-csp	91.07%	95.01%	94.69%	65.19%	93.00%	18.24 M
Faster-RCNN	92.81%	**97.68%**	**97.78%**	76.48%	95.18%	56.67 M
Ours	**97.33%**	97.01%	97.38%	**79.39%**	**97.17%**	7.01 M

Compared to other lightweight models such as YOLOv5n, YOLOv12n, and YOLOv6n, our improved YOLOv8n model demonstrates significant enhancements across all performance metrics: approximately 3.5% improvement in Precision, nearly 3% in Recall, about 2% in F1-score, around 2% in mAP@0.5, and nearly 6% in the most stringent evaluation metric mAP@0.5-0.95. The substantial gains in both mAP@0.5-0.95 and Recall validate the model’s enhanced robustness against interference in complex weather conditions.

When compared with larger lightweight models such as YOLOv7-tiny, YOLOv5-csp, and YOLOv4-csp, our improved YOLOv8n model also demonstrates clear advantages, particularly achieving nearly 10% improvement in mAP@0.5:0.95. This reflects the limited capability of existing lightweight models of comparable scale under complex weather conditions, while further demonstrating the overall superiority of the proposed model in such environments.

**Fig 12:**
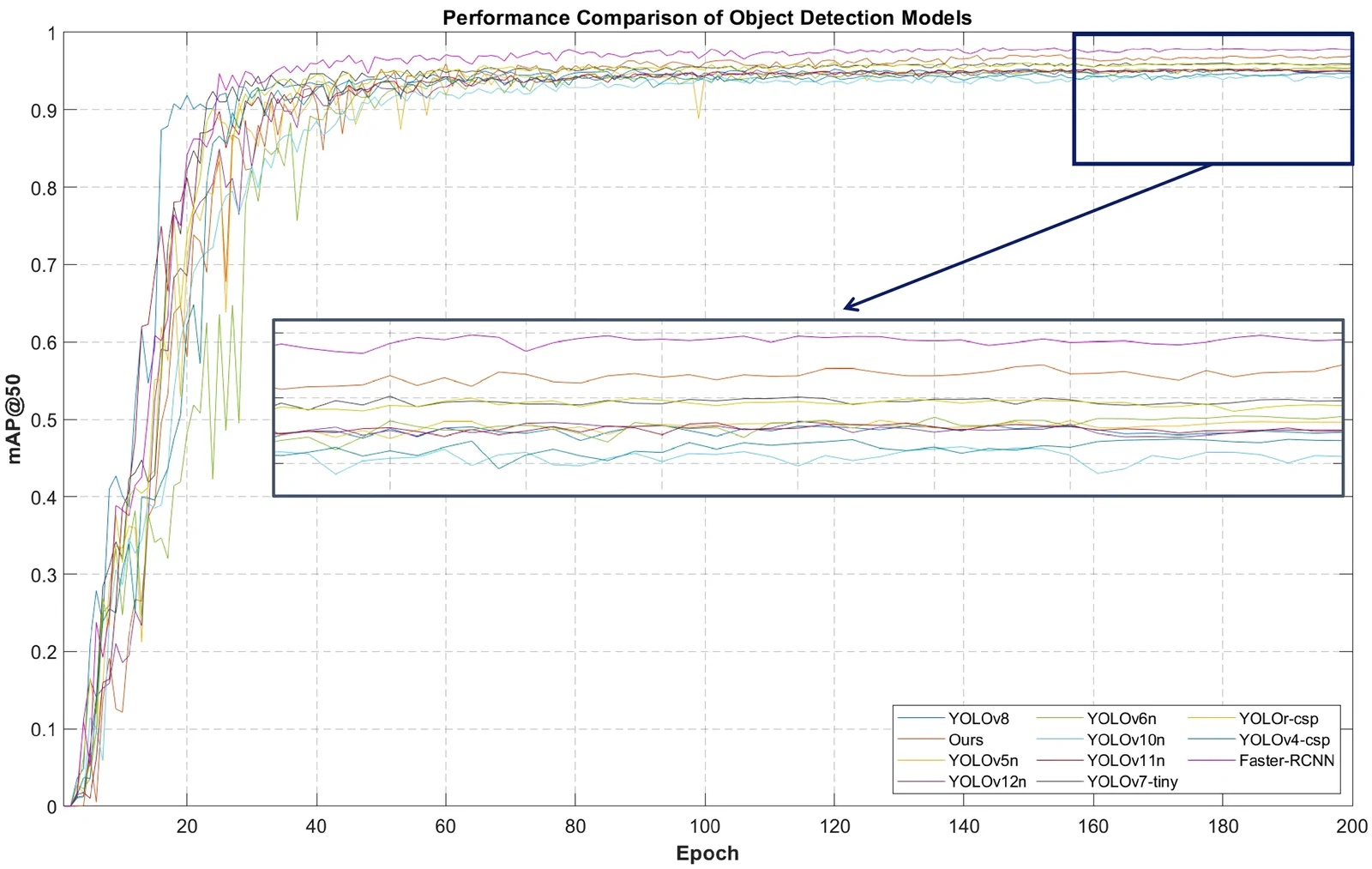
Comparison of mAP@0.5.

**Fig 13:**
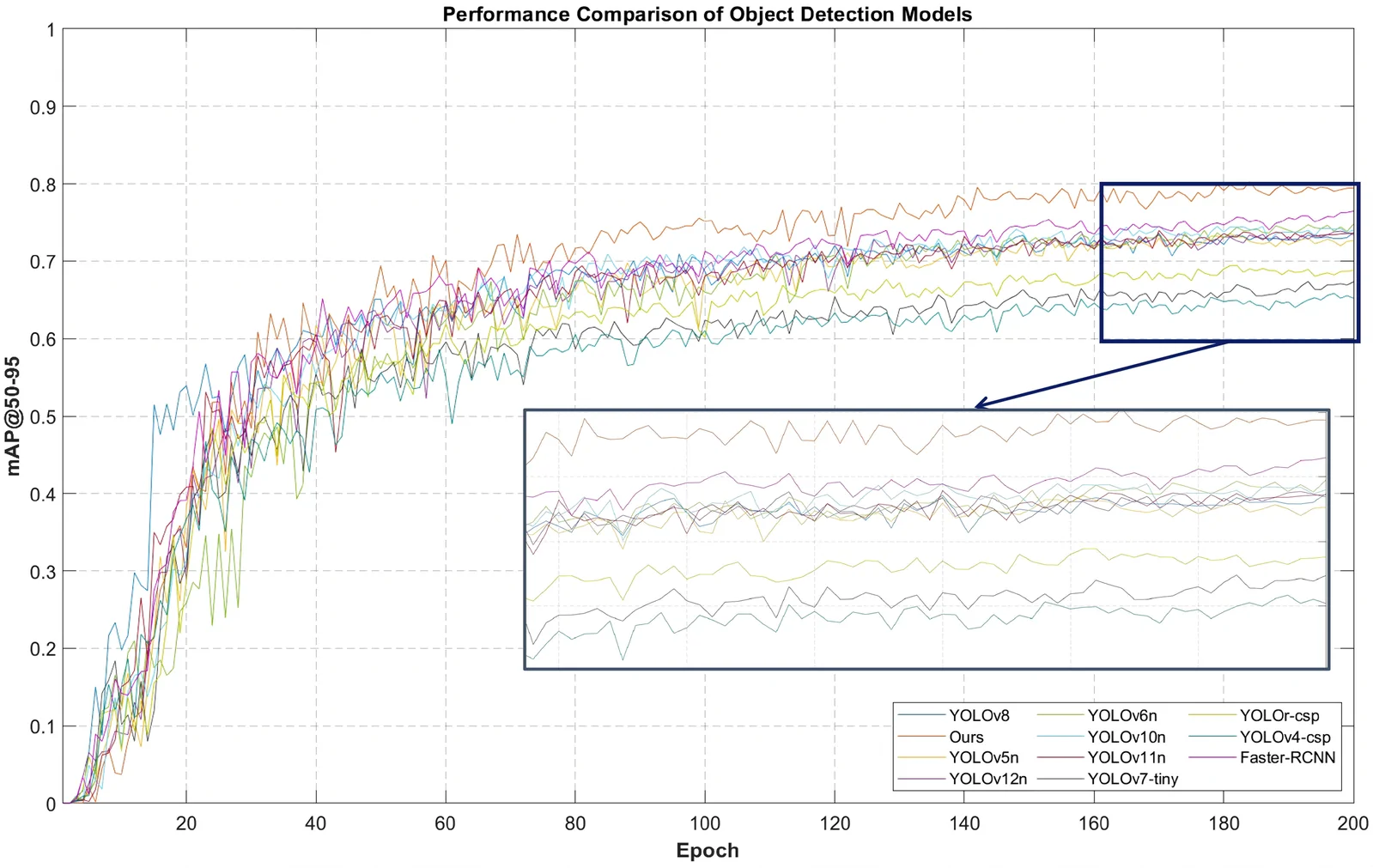
Comparison of mAP@0.5-0.95.

In comparison with the substantially larger Faster-RCNN model, although our model shows slightly lower performance in Recall and mAP@0.5, it maintains clear advantages in both Precision and mAP@0.5-0.95. This performance advantage becomes particularly noteworthy given that the Faster-RCNN model has approximately eight times the parameter count of our proposed model. As shown in Fig 12 and Fig 13, the comparisons of each model on mAP@0.5 and mAP@0.5-0.95 are presented.

### 5.3. Comparison of visualization results

**Fig 14:**
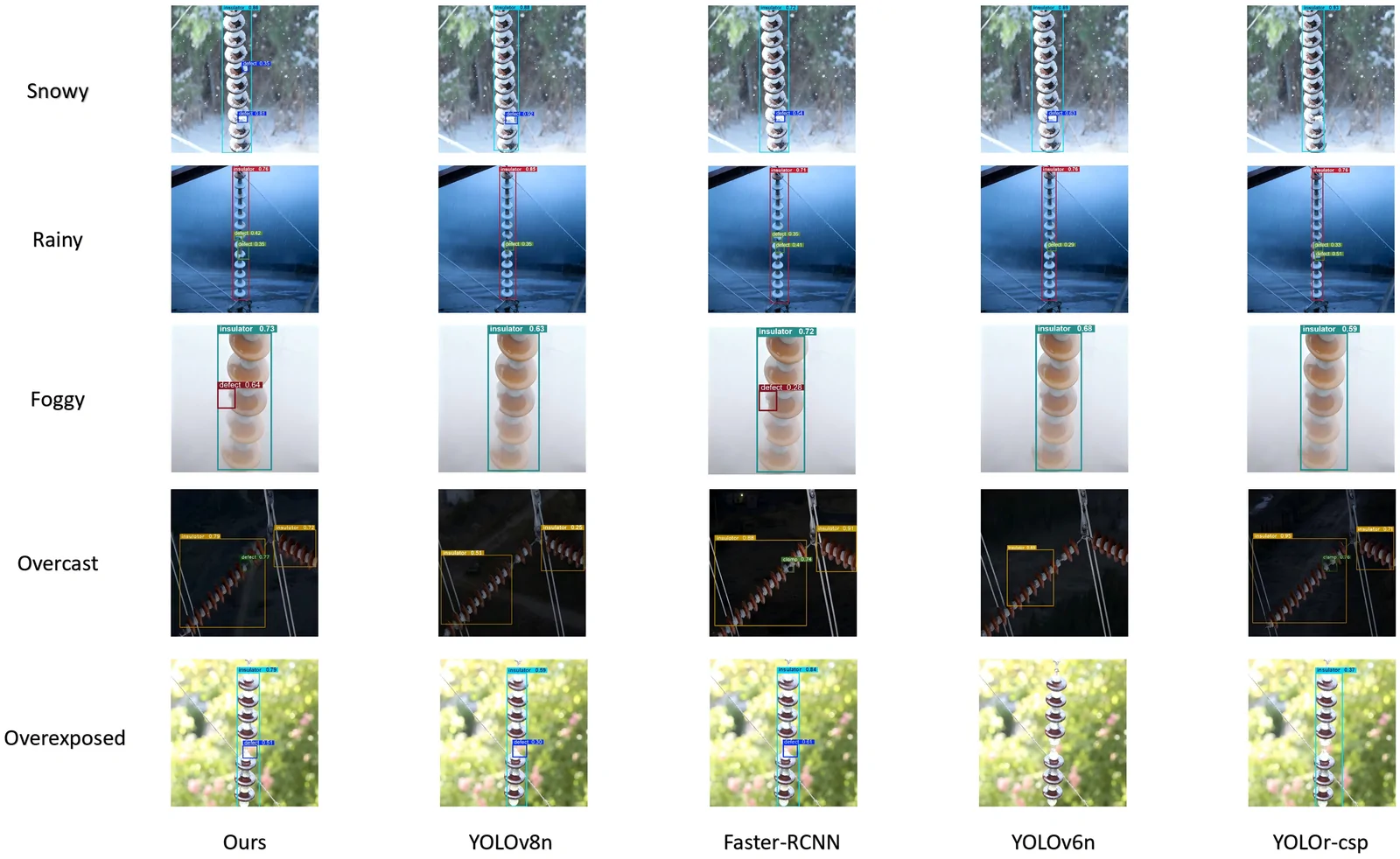
Practical application of different models in complex weather conditions.

To illustrate detection performance under different weather disturbances, five images representing snow, rain, fog, cloudy, and exposure conditions were selected from the independent test set according to predefined criteria. Each image contained complete annotations, typical weather interference, and identifiable defects, and none was selected based on favorable results for any model. All models were evaluated using the same five images. For readability, Fig 14 presents only the baseline YOLOv8n, the representative lightweight one-stage YOLOv6n, the larger CSP-based YOLOR-CSP, the two-stage Faster R-CNN, and the proposed model.

Under snowy conditions, the original image contains two insulator damage defects: one is relatively obvious while the other is less prominent due to background interference from snow. Our model is the only one that successfully detected both defects without any missed detections. In rainy conditions, there are three insulator damages in the original image. Although our model generated only two detection boxes, the box with a confidence score of 0.35 encompasses two defects, thus achieving zero missed detections. For foggy conditions, the image contains two insulator damages. The significant background interference caused by fog prevented all algorithms from completely identifying all defects; however, our model successfully detected one defect with the highest confidence score. Under overcast conditions with low ambient brightness that obscure insulator features, there is one self-shattered insulator among two insulators. Both the Faster-RCNN model and our model successfully identified all defects and insulators. In overexposed conditions with one self-shattered insulator defect, the bounding box generated by our model fits the insulator more accurately compared to other models, accompanied by a higher confidence score.

Through comparative analysis, our model demonstrates the best overall performance across these five practical scenarios, effectively handling interferences caused by various complex weather conditions. This validates the superiority of the improved model for insulator defect identification in transmission lines under challenging weather environments.

### 5.4. Edge device test results

To further validate the effectiveness of deploying the proposed scheme on UAV edge devices, the NVIDIA Jetson Orin NX (16 GB) was selected as the edge computing platform. At an input size of 640 × 640 and a batch size of 1, all models were warmed up for 50 runs and timed over 500 synchronized runs. The proposed model has a size of 7.01 MB, achieves 208 FPS, and requires 4.3 ms per frame, satisfying the deployment requirements of ≤10 MB, ≥ 30 FPS, and ≤33 ms latency. The device operates on Ubuntu 22.04 with JetPack 5.12.

Edge-device testing included YOLOv8n, YOLOv5n, YOLOv12n, Faster R-CNN, and the proposed model, representing the baseline, a typical lightweight model, a recent lightweight model, a two-stage detector, and the proposed method, respectively.

The models trained on the PC were deployed to the Jetson Orin platform. Subsequent experiments were conducted to evaluate key performance metrics, thereby further substantiating the viability of the proposed approach. The results of the edge deployment are shown in Table 5 below.

**Table 5:** Edge deployment results.

Model	Precision	Recall	map@0.5-0.95	FPS	Delay
YOLOv8n	94.96%	96.01%	74.33%	185	5.4ms
YOLOv5n	94.72%	96.82%	73.59%	**238**	**4.2ms**
YOLOv12n	93.88%	95.73%	74.28%	172	5.8ms
Faster-RCNN	93.09%	**98.23%**	77.61%	19	52ms
Ours	**97.71%**	98.03%	**80.35%**	208	4.3ms

Experimental results demonstrate that when deployed on edge devices, the proposed model achieves further improvements in accuracy metrics, outperforming comparative models such as YOLOv8n, YOLOv5n, YOLOv12n, and Faster R-CNN. Specifically, our method attains a detection speed of 208 FPS with a latency of only 4.3 ms, delivering superior detection performance while maintaining high real-time capability. Overall, the proposed approach strikes an optimal balance among detection precision, recall, and real-time processing. These findings confirm its suitability for UAV edge deployment scenarios demanding both high efficiency and low latency, thereby further validating the effectiveness and feasibility of the proposed scheme.

## 6. Conclusion

1) To address the challenges of background interference, insufficient feature extraction, and the need for model lightweighting in image-based defect detection of transmission-line insulators under complex weather conditions, an insulator defect detection method based on an improved YOLOv8n model was proposed. MobileNetV4 was introduced as the backbone network to reduce backbone complexity. The convolutional block attention module (CBAM) was incorporated to enhance the model’s sensitivity to small targets and critical regions. In addition, an ABIFPN neck architecture was designed to improve the quality of multiscale feature fusion and strengthen resistance to background interference. The SIoU loss function was further employed to improve bounding-box regression accuracy.
2) On the mixed-weather test set, the final model achieved a precision of 97.33%, a recall of 97.01%, an F1-score of 97.17%, an mAP@0.5 of 97.38%, and an mAP@0.5:0.95 of 79.39%. Compared with the baseline model, these metrics increased by 2.43, 2.32, 2.38, 2.43, and 5.47 percentage points, respectively. During testing on the Jetson Orin NX platform, the model achieved an inference speed of 208 FPS, a latency of 4.3 ms, and a weight-file size of 7.01 MB. Although its inference speed was lower than the 238 FPS achieved by YOLOv5n, it delivered higher detection accuracy, thereby achieving a favorable overall balance between accuracy and real-time performance.
3) The overall comparative results and five representative examples under typical weather conditions demonstrate that the proposed model has promising application potential on the current mixed-weather dataset. However, the available aggregate metrics and limited number of visualized examples are insufficient to establish that the model possesses equally strong robustness under rainy, snowy, foggy, overcast, and varying-exposure conditions. Therefore, the conclusions of this study are restricted to the current dataset and experimental settings.
4) This study still has several limitations. Some weather-condition samples were generated using data augmentation or synthetic methods, whereas real-world extreme weather conditions and non-weather-related degradations, such as motion blur, lens contamination, and complex occlusion, were insufficiently represented. In addition, some flight and camera metadata were unavailable during data acquisition, while class imbalance and annotation errors under low-visibility conditions may still have affected the results. Epoch-wise curves from repeated experiments were not fully recorded, nor were the power mode, operating frequency, hardware utilization, temperature, and peak memory consumption of the edge device. Future work will expand the real-world multi-weather UAV dataset; separately report precision, recall, mAP, false-positive rate, and false-negative rate for rainy, snowy, foggy, overcast, and varying-exposure conditions; provide means with standard deviations or confidence intervals; and conduct standardized evaluations of inference speed, power consumption, and memory usage across a broader range of models and edge-computing platforms.
